# Evaluation of a Custom-Developed Computer Game to Improve Executive Functioning in 4- to 6-Year-Old Children Exposed to Alcohol in Utero: Protocol for a Feasibility Randomized Controlled Trial

**DOI:** 10.2196/14489

**Published:** 2019-10-08

**Authors:** Jacobus Gidion Louw, Leana Olivier, Sarah Skeen, Alastair van Heerden, Mark Tomlinson

**Affiliations:** 1 Foundation for Alcohol Related Research Cape Town South Africa; 2 Department of Psychology Stellenbosch University Stellenbosch South Africa; 3 Institute for Life Course Health Research, Department of Global Health Faculty of Medicine and Health Sciences Stellenbosch University Cape Town South Africa; 4 Human and Social Development Human Sciences Research Council Pietermaritzburg South Africa; 5 MRC/WITS Developmental Pathways for Health Research Unit Department of Paediatrics Faculty of Health Science University of the Witwatersrand Johannesburg South Africa; 6 School of Nursing and Midwifery Queens University Belfast United Kingdom

**Keywords:** protocol, fetal alcohol spectrum disorders, FASD, cognitive dysfunction/prevention and control, executive function, experimental games, brain/drug effects, child development

## Abstract

**Background:**

Fetal alcohol spectrum disorder (FASD) is one of the most common causes of preventable intellectual disability, and the key associated deficits are in executive function (EF). Aspects of EF can be improved using cognitive training interventions. The highest prevalence of FASD globally (at a rate of 135.1 per 1000) has been found in a South African population in the Western Cape province. There is a shortage of specialized health service personnel, and there are limited remedial services. Computer-based cognitive training, if age and culturally appropriate, could be an effective way to provide the interventions with minimal need for skilled personnel and other resources. The Foundation for Alcohol Related Research has developed such a program for the South African context.

**Objective:**

This protocol aimed to evaluate whether it is feasible to use computerized cognitive training in a resource-poor context to improve cognitive function in children exposed to alcohol in utero.

**Methods:**

We are conducting a randomized controlled trial in the Saldanha Bay Municipal area, evaluating a custom-developed cognitive training program to improve the cognitive function of children aged between 4 and 6 years who were exposed to alcohol in the prenatal stage. Participants will be recruited from local Early Childhood Development centers. Community workers will interview biological mothers to identify alcohol-exposed pregnancies. Alcohol-exposed children will be randomized into an intervention or a control group of 40 participants each using block randomization. A group of 40 children not exposed to alcohol will be included in a normative group using individual randomization. The intervention group will play the game for 6 months (40 sessions). Normative and control groups will receive no intervention. Neurodevelopmental assessments will be done at baseline and upon completion of the study with all participants.

**Results:**

The intervention has started, and all baseline assessments have been done at the time of submission.

**Conclusions:**

This study will provide insight into whether computerized cognitive training is viable and effective in the South African context. It has the potential to provide a means of intervention globally and in other resource-poor context and expand the knowledge base regarding executive functioning and FASD. This paper presents the research protocol and intervention design of the study.

**Trial Registration:**

ISRCTN Registry ISRCTN17244156; http://www.isrctn.com/ISRCTN17244156.

**International Registered Report Identifier (IRRID):**

DERR1-10.2196/14489

## Introduction

### Background

Consuming alcohol during pregnancy is one of the most common causes of preventable intellectual disability [1]. The teratogenic effects of alcohol can lead to a range of physical and intellectual difficulties and disabilities, grouped together under the term fetal alcohol spectrum disorders (FASDs) [2]. To date, the highest prevalence of FASD globally has been found in selected communities in South Africa, with a prevalence rate of 135.1 per 1000 in a particular population in the Western Cape province and 119.4 per 1000 in the Northern Cape provinces [3-5].

Deficits in both elementary and higher order intellectual functioning have been found in FASD. It has been established that some of the key deficits in children are in the domains of attention, information processing, and executive function (EF) [6]. The deficits found in attention are associated specifically with encoding (temporarily holding information in memory to perform mental operations on it) and shifting (flexibly moving attention between different stimulus dimensions), along with deficits in executive control [7,8]. The EF that is found to be affected includes inhibitory control, cognitive planning, and working memory [7,9].

### Cognitive Training

Dysfunctions in EF can have serious consequences and have been associated with poor school readiness, addiction, conduct disorder, and lower educational outcomes [10]. Owing to the deficits in EF, individuals with FASD struggle with integrating knowledge and basic cognitive processes required for complex tasks [7]. Intervention in these areas is crucial as deficits have an impact on social functioning, adaptive functioning, and the ability to live independently. It has been shown that aspects of EF can be improved using training and practice [11]. Interventions include physical activities such as martial arts and yoga, improved school curricula, and computerized cognitive training [11,12]. Cognitive training has shown to have an impact on task performance and on fluid intelligence (Gf) tests [13-15]. Training on specific cognitive tasks induces neuroplasticity, and although the specific mechanisms are still unclear, it is generally accepted that training impacts synaptogenesis and neurogenesis [16]. Evidence that these mechanisms can improve the brain’s functioning has been found in populations including individuals with traumatic brain injury [17], individuals with unspecified mild or moderate mental retardation [18], and children with FASD between the ages of 6 and 15 years [19].

The impact of the improvement in specific functions is not yet clear, and results of studies examining this have been mixed [14,20]. The key question in this regard is whether training can have an impact on fluid intelligence and therefore global intellectual functioning. Although the idea that training can impact Gf has been challenged [15], there is some evidence to support it [21-23]. The sequela of the cognitive deficits associated with prenatal alcohol exposure (PAE) arguably have the most severe impact on life outcomes [24,25]. Early diagnosis and intervention can improve expected life outcomes [26]. Yet, in South Africa, an acknowledged lack of specialized health services and personnel [27] means that few affected children will be diagnosed early, and those that are diagnosed will likely not have access to any remedial programs or intervention.

### Cognitive Training in Resource Poor Contexts

Cognitive training interventions do not translate easily into a resource-poor context, and even physical activities such as martial arts and yoga require a trained interventionist. Computer-based cognitive training, if age and culturally appropriate, could be an effective way to provide the interventions with minimal need for skilled personnel and other resources. Unfortunately, existing games such as cognitive carnival or Caribbean quest [19] and other brain training games require a level of computer literacy not routinely found among low-socioeconomic status (SES) children owing to low levels of exposure to computers [12]. The game, Caribbean Quest, for example, requires children to use a keyboard and mouse. These skills would have to be taught to the children, and in some settings may serve as a confounder when measuring game performance. Participants in the Caribbean Quest study also had one-on-one sessions where they were taught meta-cognitive skills. Although this proved to be effective, in a resource-poor context, this would not be viable on a large scale [19].

In comparison with the Canadian study [9], children diagnosed with FASD in South Africa live in social environments different from those in Canada and the available resources are scarce. Participants in the cited cognitive training studies were either already receiving other rehabilitation services [17,18] or the intervention went further than only providing the cognitive training game [19]. Therefore, the question remains whether computerized cognitive training can be adapted for a resource-poor context and whether it will still have an impact.

One of the main differences between the 2 contexts is the access to personal computers and laptops. To develop games for personal computers and laptops will therefore likely exclude many participants in low-SES communities. The required skills to use these devices would likely also be lacking in these communities. Moving development to mobile computing devices mitigates these complications. There may of course be a lack of familiarity with mobile devices as well, but the more intuitive touch screen interfaces will be easier to master for young children. A move to mobile computing can also benefit children in more developed countries as well, as their first exposure to technology will likely also be in the form of mobile devices or tablet computers [28]. There are of course challenges involved with mobile computing with more issues with input lag, latency, and less memory and processing power. The benefits do outweigh the complications.

Over the past 3 years, the Foundation for Alcohol Related Research (FARR) has been developing and piloting such a cognitive training program tailored to the South African context. The intervention is designed for eventual use on tablets or smartphones that are becoming more ubiquitous even in low-income communities [29]. The FARR game is focused on exercising EFs, particularly attention, inhibition, and working memory. From the outset, it was designed to be suitable for children with little to no computer literacy, to need minimum support and intervention from professionals, and have the possibility to be scaled up as an intervention.

## Methods

### Overview

We are conducting a feasibility randomized controlled trial (RCT) to evaluate the use of a custom-developed cognitive training program to improve the cognitive function of children aged between 4 and 6 years who were exposed to alcohol in the prenatal stage compared with a control group.

We hypothesize the following:

1. Post intervention, the intervention group will score higher on psychometric assessments of EFs than the control group.
2. At baseline, alcohol-exposed children (both intervention and control groups) will score significantly lower on psychometric assessments of EFs compared with nonalcohol-exposed children (normative group).
3. Post intervention, the intervention group will score higher on psychometric assessments of EFs than the control group, but lower than the normative group.
4. Post intervention, the intervention group will show greater improvement on psychometric assessments of EFs than the control and normative groups.
5. Improvement in game performance will be correlated with improvement on psychometric assessments of EFs.
6. Improvement in psychometric assessments of EFs will be positively correlated with total time spent playing the game.

### Research Ethics and Approval

Ethical approval for the protocol has been obtained from the Health Research Ethics Committee at Stellenbosch University (reference number: N16/05/063). The Standard Protocol Items: Recommendations for Interventional Trials (SPIRIT) were used in designing the study and writing of the protocol. A checklist and populated checklist have been provided as per the recommendations. The trial has been registered with the ISRCTN registry (ISRCTN17244156). Should the intervention prove successful, the game will be made available to all participants. This would, however, require the participant to have access to a suitable device as providing participants with tablet computers would not be feasible.

### Setting

The RCT will be conducted in the Saldanha Bay Municipal (SBM) area in the Western Cape province of South Africa. Saldanha Bay is the largest natural port in Africa, most of the economic activity in the area is related to fishing, agriculture, and iron exports. There is, however, significant income inequality and an unemployment rate of 17% [30]. FARR has previously conducted a prevalence study in this area, where a prevalence rate of FASD of 64.2 per 1000 was found [31]. FARR subsequently implemented a comprehensive prevention and awareness program between 2013 and 2016 and is currently running an awareness program in the SBM area.

Although the aim of the intervention is to develop cognitive training for children with FASD, it is not possible to identify and diagnose a large group of children with FASD within the study’s timeframe. The intervention will thus be evaluated using children who were exposed to alcohol during pregnancy regardless of whether they have received a diagnosis of FASD. To screen for alcohol exposure, we will conduct in-depth interviews with mothers of children who attend Early Childhood Development (ECD) centers in the SBM area.

### Eligibility Criteria

#### Inclusion Criteria

Children aged between 4 and 6 years will be eligible for inclusion. Children must reside in the SBM area and attend or be enrolled in an ECD center.

#### Exclusion Criteria

Children with a physical disability that will hamper their interaction with the program will be excluded, for example, severe eyesight problems.

### Intervention

The efficacy of cognitive training is influenced by the time spent interacting with the program and the difficulty of the tasks presented, especially in terms of whether skills that are not directly trained will improve [18]. In the first step, the specific processes to be targeted were identified using the available literature. In consultation with a software developer, the basic interface and game mechanics were designed, focusing on an intuitive interface and game mechanics that will allow the same interaction to be used with all tasks. In the alpha (initial) version, it is possible to select different tasks to ensure data can be gathered for all stages and tasks. Data that are logged will include the date and time of the play session, the response time per item, and whether the correct response was selected for each item.

The theoretical basis for the game design involves 2 different definitions of *scaffolding* in 2 different contexts. The first refers to work on childhood development and cognitive development. In this context, scaffolding refers to the support of cognitive development by reducing a complex problem to subproblems, and by solving the subproblems, the ability to solve the complex problem is gained [32]. The game was developed to incrementally increase the demands on various cognitive abilities, with continued successes on one level of difficulty eventually enabling success on a higher level of difficulty.

The second context of scaffolding refers to neuroplastic scaffolding. This term is generally used in connection with age-related cognitive decline [33], but as the target population of this intervention frequently suffers from structural brain abnormalities [2], the same concept of neuroplastic scaffolding should still apply. It refers to compensatory neural activity aimed at supporting damaged, inefficient, or poorly functioning cognitive functions. Scaffolding happens through recruitment of additional prefrontal cortex activity, neurogenesis (formation of new neuronal connections), and distributing cognitive processing over various brain structures. This theory supports the hypothesis that practice and training can enhance the process of scaffolding [34]. This was the driving principle behind the conceptualization and design of the game.

To try and ensure long-term engagement with the game, meta-game elements have been added to the game in the form of a progress bar and animations that only appear once you have completed a set number of tasks. There will be no external rewards or incentives for playing. This is intentional as a key design consideration is that it should be fitting for context where no external rewards would be available. Tasks were designed to require effortful use of inhibitory control, cognitive planning, set shifting, and working memory for completion. The tasks have been designed in such a way that regardless of which function is being targeted, the interface and interaction with the participant does not change. If the interaction remains the same, it is easier to switch seamlessly between stages to maintain optimum difficulty.

The game we have designed (1) is easy to use regardless of computer literacy, (2) logs performance on various metrics (response times and error rates), (3) has continuously adaptive difficulty levels to maintain a suitable level of challenge, and (4) will be open access (free to distribute). Before making the intervention available, it is necessary to test whether it does in fact have an impact on cognitive development.

An alpha version of the program was piloted on a sample of normally developing children to ensure that the tasks escalate in difficulty. This provided an opportunity to ensure that the timing of visual stimuli is age appropriate for the proposed sample’s age. The pilot also provided normative data on participant performance to guide further development of the program.

#### Control Condition

Children in the control group will not be receiving any intervention. They will be assessed at the start of the study and there will be no further interaction with project staff or community workers until the intervention has run its course and they receive the follow-up assessment.

### Recruitment

Trained community workers will contact mothers through the ECD centers. They will obtain informed consent to conduct a maternal interview and to include their child in the study in one of the 3 groups. The interviews will include demographic questions and information on alcohol use during pregnancy. The interview questionnaire has been used extensively in prevalence studies in South Africa to identify whether children were exposed to alcohol during pregnancy [3,31-33]. The interview will indicate if a mother consumed alcohol during pregnancy, and it will give an indication of the amount of alcohol consumed. If a child was exposed to more than 3 units of alcohol in 1 drinking session, they will be classified as *alcohol exposed*. This is in line with the latest FASD diagnostic guidelines [2].

As alcohol-exposed children are identified, they will be allocated to the intervention or control group using block randomization with a block size of 8. The randomization will be done by the primary investigator using assigned study numbers to blind them to the participants’ identity. Once a block has been filled, the 4 participants allocated to the intervention will start the intervention. The normative group will be recruited from children not exposed to alcohol during pregnancy on the basis of the maternal interviews using random number tables. A list of study numbers will be created and entries in the list will be selected based on the tables [35]. The primary investigator will be blind to participants’ identities during this operation.

### Data Collection

The maternal interview will be conducted by community workers. They have extensive experience in conducting this particular interview as they interviewed mothers during previous prevalence studies using the same tool. Any additional community workers required will be trained by the primary investigator. After the training, they will conduct mock interviews with their fellow community workers.

The neurodevelopmental assessments will be conducted by a psychometrist with experience in assessing by using the NEuroPSYchological Assessment, Second Edition (NEPSY-II). The psychometrist will also have extensive experience working with children exposed to alcohol during pregnancy and have participated in FASD prevalence studies.

The intervention will be overseen by trained community workers. They will be trained on how the game should be played and instructed on how they should interact with the children after they have mastered playing the game on their own. The community workers will also be trained on how to copy and secure the game logs kept on the tablet computers.

### Study Procedures

**Figure 1:**
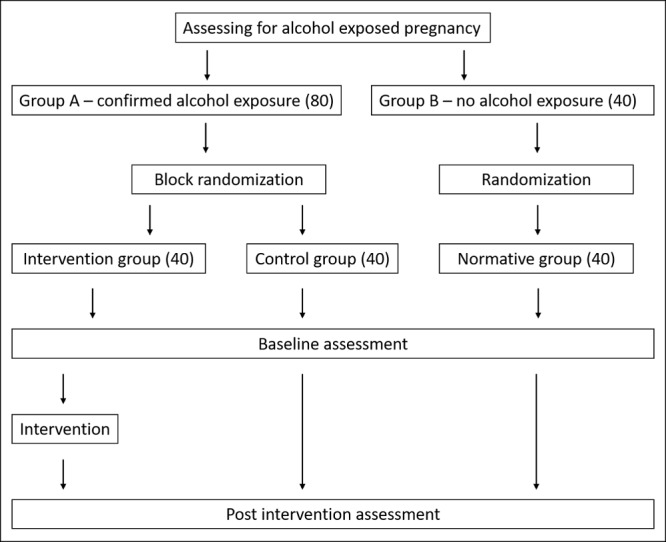
Overview of study design.

Alcohol-exposed children, as assessed by maternal interviews, will be randomized into an intervention or a control group with 40 participants in each group, using block randomization. The maternal interview will contain questions regarding alcohol use just before and during pregnancy, including the number of standard units of alcohol consumed, with 3 standard units being the threshold for determining alcohol exposure. A group of 40 unexposed children will be randomized in a group to provide normative data using individual randomization. An overview of the study design is shown in Figure 1. As the participants are drawn from a low-SES and resource-poor environment, the performance of the normative group will serve as a more suitable gauge of cognitive development in the participants’ specific contexts. This will enable us to better interpret the results of the neurodevelopmental assessments.

The SPIRIT schematic is shown in Table 1. Baseline assessments examining cognitive function will be conducted with all 3 groups. The intervention group will then play the FARR game twice a week for 6 months (40 play sessions in total). Post intervention, all 3 groups will receive follow-up cognitive assessments. Data gathered by the game will also be collected from the intervention group. All participants will start the intervention at the same level of difficulty. They will be given the opportunity to play the game during hour-long sessions facilitated by community workers. Play will, however, be self-directed; participants will be encouraged to play but will be allowed to stop at any time during a session. As participants log into the devices, they will be able to continue at the difficulty level they had previously reached.

There are 4 different stages in the game of increased complexity. After 10 trials in a stage, a participant automatically moves on to the next for 10 trials. After the fourth stage, participants start at the first stage again. The difficulty of each stage varies independently based on a participant’s performance. Getting 8 or more correct increases difficulty, getting fewer than 4 correct decreases difficulty, and otherwise the difficulty remains the same.

**Table 1:** A trial schedule per the Standard Protocol Items: Recommendations for Interventional Trials schematic.

Timepoint		Study period			
		Enrollment	Allocation		
		−*t*_1_	0	*t*_1_	*t*_x_

**Enrollment**					
	Eligibility screen	X	—^a^	—	—
	Informed consent	X	—	—	—
	Maternal Interview	X	—	—	—
	Allocation	—	X	—	—
**Interventions**					
	FARR^b^ game	—	—	X	—
	No intervention control group	—	—	X	—
	No intervention normative group	—	—	X	—
**Assessments**					
	NEPSY-II^c^ psychometric assessment	—	X	—	X
	Data logged by FARR game	—	—	—	X

^a^Not applicable.

^b^FARR: Foundation for Alcohol Related Research.

^c^NEPSY-II: NEuroPSYchological Assessment, Second Edition.

### Measures

The primary outcome is EF, as measured using the NEPSY-II psychometric assessment score. The secondary outcomes are (1) game performance, as measured in the game log files, (2) reaction time, and (3) increase in the level of difficulty of the task.

Additional demographic data will be gathered during the recruitment process. In addition to alcohol use, data will be gathered on age, gravidity, parity, years of schooling, and household income.

All participants will be evaluated using a selection of subtests of the NEPSY-II that measure executive functioning and working memory. Cognitive function, focusing on EFs, will be measured using the NEPSY-II [36]. The NEPSY-II is a compendium of tests based on the Luria theoretical approach to neurological assessment. It is individually administered and has been shown to be successful in diagnosing a range of childhood disorders. The subtests of this test can also be selected based on the specific domains to be tested [37].

Scores obtained on the subtests of the NEPSY-II are compared with the scores of a normative group based on age. The normative group of the NEPSY-II was selected to closely match the US population between 3 and 16 years of age [38]. This group will differ significantly from the populations of interest in this study; however, the NEPSY-II is relatively insensitive to language and culture differences. Although the results need to be interpreted cautiously, the test remains clinically useful [39]. Insensitivity to language is an important feature as the study sample will be drawn from a predominantly Afrikaans-speaking community, which could affect test performance [40].

In the standardization of the NEPSY-II, subtests showed adequate-to-high internal validity. There was no significant practice effect with re-administration of the test in a short space of time (around 3 weeks), supporting its use for both the pre- and postintervention assessments in this study [41]. Inter-rater agreement was high on both the objectively and more subjectively scored test, and the reliability of subtests remained stable [38]. The inclusion of the third group of children not exposed to alcohol in pregnancy will help guide the interpretation of results.

The NEPSY-II uses separate batteries for children aged 3 to 5 years and children aged 6 to 16 years. Between the ages of 3 and 5 years, not all subtests can be administered. We will be focusing on the subtests that are the same for all participants from 4 to 6 years. Participants who are aged 6 years at baseline and/or at the postintervention assessment will be tested on 4 additional subtests to provide a broader base for comparing game tasks and EFs (see Table 2).

**Table 2:** Subtests to be assessed.

Domain	Attention and executive function	Language	Memory and learning
All ages	Statue	Comprehension of instructions	Memory for designs
All ages	—^a^	—	Narrative memory
All ages	—	—	Sentence repetition
6-year-olds only	Auditory attention	—	—
6-year-olds only	Design fluency	—	—
6-year-olds only	Inhibition	—	—
6-year-olds only	Inhibition naming	—	—

^a^Not applicable.

#### Game Log Files

Additional data will be obtained from the log files generated by the game. The file will record error rates and response times for all tasks. Each child will be allocated a numbered tablet or a profile on a specific tablet, depending on what proves the most practical, and the log file will then be associated with their study number.

#### Sample Size

A total of 120 participants will be recruited and randomized to one of 3 study arms. This is in line with the sample sizes of previous studies looking at FASD and cognitive functioning [9,19,39]. Previous studies of this nature showed a medium effect size [42-45]; however, owing to the differences in method and there not being interventionists involved in the training sample, size calculations were done for a small (*d*=0.2) effect size. With a significance level of .05, 120 participants in 3 groups would yield a statistical power of 0.79 for the repeated measures multivariate analysis of variance (MANOVA) comparing the performance between the 3 groups.

There are approximately 46 ECD centers, and in SBM area, 1593 children aged between 3 and 5 years have enrolled at these centers and 1635 6-year-olds have enrolled in Grade R [46]. Assuming a prevalence rate of 24% of consuming alcohol during pregnancy on the basis of a prevalence study conducted in SBM area [31], it should be possible to reach the desired sample size through 400 interviews.

### Data Analysis

For the primary hypothesis, the postintervention scaled scores of the intervention group will be compared with those of the control group. This will be done using a MANOVA with group membership as the predictor variable and the scaled scores of the 5 NEPSY-II domains (see Table 1) as outcome variables. Discriminant analysis will be done on the outcome of the MANOVA with the domain scores as predictor variables and group membership as outcome variables.

During further analysis, the baseline NEPSY-II scaled scores of the intervention and control groups will be pooled and compared with the scores of the normative group. This will also be done using a MANOVA with group membership as the predictor variable and the scaled scores of the 5 NEPSY-II domains (see Table 1) as outcome variables. Discriminant analysis will again be done on the outcome of the MANOVA with the domain scores as outcome and group membership as predictor variables.

Postintervention NEPSY-II scaled scores (see Table 1) of the intervention, control, and normative groups will be compared using a MANOVA. Group membership will be the predictor variable, and domain scores will be the outcome variables. Discriminant analysis will be conducted with the domain scores as outcome variables and group membership as predictor variables.

Difference scores between baseline and postintervention assessments will be calculated for all participants. These scores will be compared using a MANOVA with group membership as the predictor variable and changes in domain scores (see Table 1) as outcome variables. Discriminant analysis will be done on the results with changes in domain scores as outcome and group membership as predictor variables.

For the secondary hypothesis, the improvement in game performance will be quantified as how many difficulty levels a participant has successfully completed in each of the 3 different stages of the game. This will then be correlated with performance in the NEPSY-II domain scores in a covariance matrix using the Pearson correlation coefficient. A separate analysis will be done including only 6-year-old children using the additional domains tested (see Table 1).

Additional exploratory analyses will be done comparing the demographic information of the 3 groups. These data will be drawn from the maternal interview. Demographic data on the mothers of the alcohol-exposed children (both intervention and control groups) will be pooled and compared with nonalcohol-exposed children to ascertain if there were significant differences between the 2 groups. This will be done using 2 tailed *t* tests for independent samples. To account for family-wise errors, Bonferroni-adjusted values will also be calculated. The variables to be analyzed are age, gravidity, parity, years of schooling, and income. Tobacco and drug use will also be compared among the alcohol-exposed and nonalcohol-exposed groups using a chi-square test.

Further exploratory analysis will be done on the game logs. Reaction time in the various stages of the game will be used to conduct an exploratory factor analysis. Looking for common variations among the various game metrics may unearth underlying variables that better explain which EFs are associated with which game tasks. For each participant, their reaction times for the highest level they successfully complete will be used. Using regularized exploratory factor analysis owing to the small sample size, we can examine whether the conceptually different tasks do in fact target different EFs.

## Results

At the time of submission, the majority of recruitment was completed, and the intervention phase had already started for some participants.

## Discussion

To the best of our knowledge, this study is the first of its kind in the South African context. Although there is evidence for the use of cognitive training games to improve executive functioning in children with FASD, the feasibility of such an approach in a resource-poor context has not been evaluated. This study will provide data on both the general approach of cognitive training games in the South African context and the feasibility of our custom-developed game. These data will guide decision making on whether the current program is suitable for the purpose of remediation, whether it needs redesign, whether the method holds promise but requires a different game or program, and finally whether computer-based cognitive training is a feasible strategy at all.

Should the trial show promising results, the next step would be to use the results of this study to inform further development. Further trials with more participants and a control group receiving some form of intervention would provide support for scaling up the intervention. In addition, the intervention can then be made available to all interested parties free of charge to provide at least some basic form of evidence-based remediation. If there are strong correlations between the NEPSY-II scores and the game tasks, further studies could evaluate whether the game could be used as a screening tool for difficulties with EFs.

As the game does not use language in any of the tasks, it can be used outside of the South African context with ease. With further development, it will also be available for more devices (smartphones) and not only for tablet computers. It could therefore be a valuable tool in other countries where remedial resources are not available, and it can be used by caregivers and parents to support existing remedial efforts. The nature of the cognitive training does not limit its use to FASD remediation. If children exposed to alcohol show improvement, theoretically, children with cognitive deficits with different etiologies will also benefit.

Regardless of the trial outcome, this study will add significantly to the literature on executive functioning in children with PAE. It will also provide more insight into how the environment and development of children with PAE differs from children who were not exposed to alcohol in utero.

## Abbreviations

ECD: Early Childhood Development
EF: executive function
FARR: Foundation for Alcohol Related Research
FASD: fetal alcohol spectrum disorder
MANOVA: Multivariate analysis of variance
NEPSY-II: NEuroPSYchological Assessment, Second Edition
PAE: prenatal alcohol exposure
RCT: randomized controlled trial
SBM: Saldanha Bay Municipal
SES: Socioeconomic status
SPIRIT: Standard Protocol Items: Recommendations for Interventional Trials
